# Assessment of Vascularity in Hepatic Alveolar Echinococcosis: Comparison of Quantified Dual-Energy CT with Histopathologic Parameters

**DOI:** 10.1371/journal.pone.0149440

**Published:** 2016-02-22

**Authors:** Yi Jiang, Jiaqi Li, Jing Wang, Hu Xiao, Tingting Li, Hui Liu, Wenya Liu

**Affiliations:** Imaging Center, The First Affiliated Hospital of Xinjiang Medical University, Urumqi, Xinjiang, China; University of Munich, GERMANY

## Abstract

**Purpose:**

To investigate whether dual-energy computer tomography(DECT) could determine the angiographic vascularity of alveolar echinococcosis lesions by comparing the quantitative iodine concentration (IC) with the microvascular density (MVD).

**Material and Methods:**

Twenty-five patients (16 men, 9 women; mean age, 40.9 ± 13.8 years) with confirmed hepatic alveolar echinococcosis (HAE) underwent DECT of the abdomen, consisting of arterial phase (AP), portal venous phase (PVP), and delayed phase (DP) scanning, in dual-source mode (100 kV/140 kV). Image data were processed with a DECT software algorithm that was designed for the evaluation of iodine distribution in the different layers (marginal zone, solid and cystic) of the lesions. The CT patterns of HAE lesions were classified into three types: solid type, pseudocystic type and ‘geographic map’ (mixed) type. The IC measurements in different layers and different types of lesions were statistically compared. MVD was examined using CD34 immunohistochemical staining of the resected HAE tissue and scored based on the percentage of positively stained cells and their intensity. Pearson’s correlation analysis was used to evaluate the potential correlation between DECT parameters and MVD.

**Results:**

A total of 27 HAE lesions were evaluated, of which 9 were solid type, 3 were pseudocystic type and 15 were mixed type. The mean lesion size was 100.7 ± 47.3 mm. There was a significant difference in the IC measurements between different layers of HAE lesions during each scan phase (p < 0.001). The IC in the marginal zone was significantly higher than in the solid and cystic components in AP (2.15 mg/mL vs. 0.17 or 0.01 mg/mL), PVP (3.08 mg/mL vs. 0.1 or 0.02 mg/mL), and DP (2.93 mg/mL vs. 0.04 or 0.02 mg/mL). No significant difference was found among the different CT patterns of HAE lesions. Positive expression of CD34 in the marginal zones surrounding HAE lesions was found in 92.5% (25/27) of lesions, of which 18.5% (5/27) were strongly positive, 62.7% (17/27) were moderately positive, and 11.1% (3/27) were weakly positive. In contrast, 7.4% (2/27) of the lesions were negative for CD34. There was a positive correlation between IC measurements and MVD in the marginal zone of HAE lesions (r = 0.73, p < 0.05).

**Conclusions:**

The DECT quantitative iodine concentration was significantly correlated with MVD in the marginal zones surrounding HAE lesions. Dual-energy CT using a quantitative analytic methodology can be used to evaluate the vascularity of AE.

## Introduction

Alveolar echinococcosis (AE) is caused by the parasitic metacestode *Echinococcus multilocularis*, which often affects the liver of various intermediate hosts, including humans[1]. Clinically, most cases of hepatic AE (HAE) appear nonspecific and difficult to differentiate from other liver diseases[2, 3] because they show the characteristics of a malignant tumor, with destructive tissue growth, invasion of adjacent organs and distant dissemination[4]. Historically, surgery has been the recommended treatment for early HAE. Long-term chemotherapy with albendazole or benzimidazoles is necessary for the majority of patients with unresectable or postoperative HAE lesions[5]. Nevertheless, related prognosis-associated follow-up parameters for AE are still lacking, and therefore, safe drug withdrawal and surgical removal, if possible, remain the gold-standard treatment for HAE[3]. The search for well-validated non-invasive markers to determine metacestode viability in vivo is clearly warranted, particularly for markers that are translatable to the clinical setting[6].

The current clinical diagnosis of HAE relies mainly on the detection and follow-up of parasite lesions by imaging methods[7]. F18-fluorodeoxy-glucose-positron emission tomography (FDG-PET) combined with CT (PET/CT) is currently considered a reliable tool for the detection of metabolic activity in AE[8]. However, complicated equipment and high cost limit the widespread utilization of PET/CT as a routine modality for HAE evaluation. This limitation is especially concerning because the major endemic areas of HAE are poor areas. Thus, other accurate, economical and practical methods are required for the evaluation of AE lesions. Even though it involves exposure to relatively high levels of radiation, multidetector CT (MDCT) is the most widely used imaging modality for the detection and characterization of known or suspected HAE in the majority of low-resource countries and districts, including China, where this disease is endemic in rather remote areas of the western provinces and autonomous regions[9]. Unfortunately, traditional imaging techniques, such as CT, do not permit the evaluation of disease progression for HAE. The detection of HAE viability remains an important challenge to radiologists.

Angiogenesis is one of the aspects of periparasitic cell-mediated immune reactions that have been most neglected in immuno-pathological studies of echinococcosis[10]. In addition, immunostaining for extracellular matrix proteins in the periparasitic areas of AE have shown neo-angiogenesis in the periparasitic granuloma, which is likely involved in the trafficking of immune cells to and from the lesions[11, 12]. This neo-angiogenesis may also explain some findings of AE imaging, such as delayed enhancement of the periphery of the lesions under a CT contrast-enhanced scan[13] and an uptake of fluorodeoxyglucose in PET images[14]. Thus, angiogenesis is an indispensable index for the evaluation of viability in AE.

In our previous study[15], dynamic contrast-enhanced CT perfusion was explored as a potential new method for assessing the response of lesion vascularization to treatment of HAE with antiparasitic drugs, and it revealed different levels of angiogenesis in the peripheral area of HAE lesions. This result has confirmed the feasibility of using modern imaging technologies to detect blood microcirculation in AE. However, some limitations, such as limited coverage of all lesion sites, unstandardized post-processing, and radiation dose, have prohibited the application of this technology in routine clinical practice[16]. In contrast to conventional CT imaging, which provides only structural and morphologic information, dual-energy CT (DECT) has emerged as a promising functional imaging modality to obtain clinically relevant images[17, 18]. By obtaining quantitative data on iodine-related attenuation (IRA, measured in Hounsfield units, HU) and iodine concentration (IC, measured in mg/ml) in selected sample tissues[16], DECT provides a more precise and accurate measurement of lesion vascularity[19]. Recently, DECT has been used as a biomarker for tumor response to treatment in a series of preliminary studies[18, 20]. One preliminary study[17] showed that IC in DECT reflects the blood perfusion changes in the vital tumor burden and is a sensitive and specific marker that represents a measurement of vascularization for the lesion. Therefore, it appears reasonable to use DECT as a non-invasive, simple, low-radiation and primary modality for imaging of HAE and investigating vascularity, which could influence therapeutic decisions.

Although there is no single standardized and authorized method to evaluate the degree of vascularization in tumors, the microvessel density (MVD) count has been used as a reliable indicator of tumor angiogenic activity by counting the number of vascular profiles in an immunohistochemically stained tissue section[21]. Researchers in oncologic studies[22, 23] have found significant correlations between imaging-derived parameters and MVD.

However, the correlation between imaging results and the complex underlying pathology of HAE has rarely been studied. A more precise depiction of the pathological basis of images that are obtained using imaging techniques would allow researchers and clinicians to better interpret the imaging aspects and character of HAE.

Thus, the purpose of the present study was to prospectively investigate whether iodine quantification of DECT could detect the vascularity of HAE lesions by comparing its results with those of histopathological angiogenic parameters. We hope that our results will contribute to improved diagnosis and follow up of patients with HAE.

## Materials and Methods

### Patients

This prospective study was approved by the institutional review board of The First Affiliated Hospital of Xinjiang Medical University. All of the patients provided written informed consent to participate in this study.

This study included patients who were diagnosed based on imaging, historical, and serological criteria in accordance with the recommendations of the World Health Organization-Informal Working Group on Echinococcosis[5] and those who were assessed as having a resectable HAE lesion so that we could obtain specimens. The exclusion criteria were having undergone a radical hepatectomy and contraindications to CT examination. During a period of 16 months between June 2013 and October 2014, 42 patients (22 men, 20 women; mean age 52.3 years from different ethnic groups, including Han, Uygur, Kazak and Tibetan) who were clinically suspected of having HAE were admitted to the liver surgery department of our hospital and included in this study. During the study, eight patients were excluded (two declined to participate in the study, four already had imaging data from another hospital, and two had abnormal renal function), and six patients left for economic reasons; three patients were followed up with chemotherapy. Thus, 25 patients (16 men, 9 women; mean age 40.9 ± 13.8 years; age range, 22–68 years) with 27 HAE lesions that were verified histologically for radical hepatectomy were identified and included. At the time of inclusion, a histologic diagnosis was made based on the tissue samples from surgical resection for all patients; 23 patients (92%) underwent partial liver resections, and two (8%) patients underwent liver transplantation. Contrast-enhanced DECT was performed less than 48 hours before the operation.

### CT Scanning Protocol

All of the abdominal CT scans were performed from the dome of the liver to the iliac crest on a second-generation dual-source DECT system (SOMATOM Definition Flash VA44A, Siemens Healthcare Sector, Berlin and Munchen, Germany) in dual-energy mode. Images were obtained during a single inspiratory breath-hold in the craniocaudal direction. For contrast-enhancement scanning, iodine-based contrast medium (Iomeprol-350, Bracco Imaging SpA, Milan, Italy) was administered via an automated dual-syringe power injector (Tennessee XD2003, Ulrich GmbH & Co. KG, Germany) in an antecubital vein at a flow rate of 4.0 mL/s followed by a 30-mL saline flush at the same injection rate. The total volume of contrast medium was fitted to the body weight using an adapted injection protocol of 1.24 mL/kg. Triphasic contrast-enhanced scanning, consisting of AP,PVP and DP was performed in all cases. The scan time for AP was determined using a bolus-tracking method in which the arrival of the contrast medium in the abdominal aorta was detected at a threshold of 120 HU, after which the scanner was launched with a 6-s delay. The PVP and DP phases were acquired 30 and 60 s, respectively, after AP examination. Automatic tube current modulation (CARE Dose 4D) was used in all cases according to the manufacturer’s recommendations. The other scanning parameters were as follows: detector collimation, 64 × 0.625 mm; rotation time, 0.5 s; pitch, 0.6; slice, 3.0 mm; and tube voltage, 140 kV and 100 kV at 100 and 210 effective mA, respectively. All of the images were reconstructed with standard soft tissue reconstruction kernels (D30f) at a 1.5-mm slice thickness in 1.5-mm increments.

### Image Postprocessing

After scanning, all of the original data were transferred to the workstation (Syngo MMWP VE40A, Siemens Medical Solutions, Berlin and Munchen, Germany). The virtual non-enhanced (VNE) images and iodine-enhanced images were made using the liver Virtual Non-Contrast (Liver VNC) application mode of dedicated dual-energy postprocessing software (Syngo Dual Energy; Siemens Medical Solutions, Berlin and Munchen, Germany). This software provides an iodine overlay image that illustrates the iodine distribution in each individual CT voxel, representing the IRA and IC [24], which were generated for evaluation. The iodine-enhanced images were superimposed onto virtual non-enhanced images to combine the iodine distribution with anatomical information.

### Image/Quantitative analysis

**Fig 1:**
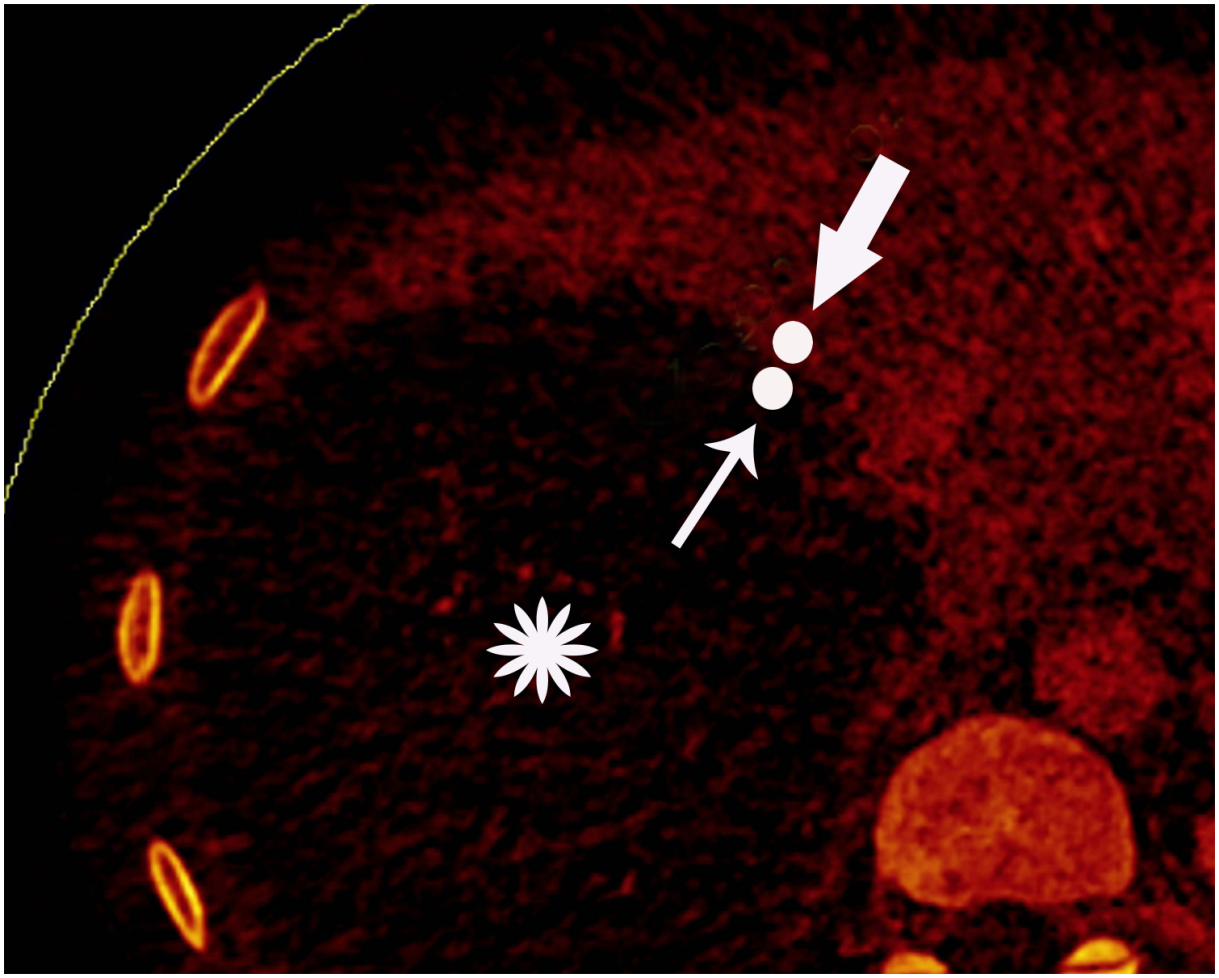
Definition of the marginal zone of HAE lesion. Displayed is an example of location with a margin area with size less than 5 mm between solid component of HAE and normal liver parenchyma exhibited on DECT contrast enhanced mixed iodine overlay images.

**Fig 2:**
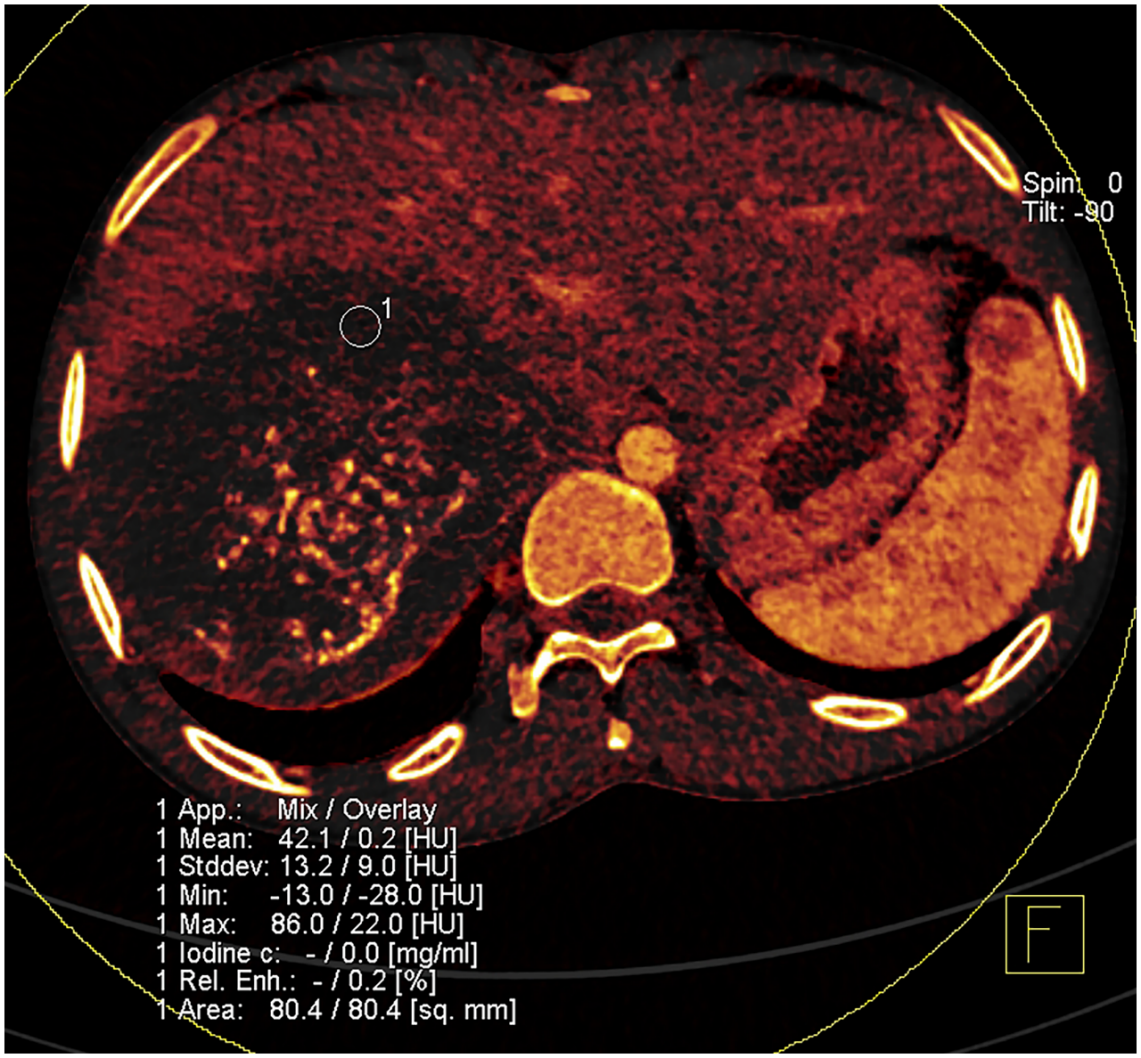
DECT image of HAE in right lober of a 42-year-old man. Iodine overlay image shows iodine distribution superimposed on virtually non-enhanced dataset. Region of interest (1) is on HAE lesion. Display includes mean attenuation measurement on virtually non-enhanced and iodine image, SD of these attenuation measurements, and iodine concentration in milligrams per milliliter. In this solid component of HAE lesion iodine concentration is 0 mg/ml.

The CT images were analyzed and evaluated by two experienced radiologists who were blinded to the clinical history of the patients. In cases of discordant interpretations, decisions on CT findings were reached by consensus. For quantitative analysis of the mean iodine-density of HAE lesions in the three contrast scan phases, the round or elliptical region of interest (ROI) was placed on all HAE lesions on an iodine overlay image (1.5-mm slice thickness), and the equivalent IC in milligrams per milliliter was determined. According to the CT images, the measurable target HAE lesions were classified into three types: solid, pseudocystic and ‘geographic map’ (mixed)[25]. First, routine CT images were evaluated to assess the calcification, cystic and solid components. CT scans were also used to evaluate the border characteristics of the lesions. Subsequently, the lesions were visualized on an iodine overlay image and the ROIs were placed separately into the different layers (marginal zone, solid and cystic) of the HAE lesion and liver, carefully avoiding interference from the surrounding major blood vessels, calcification and bile duct tissue, where volume averaging can occur. According to previous studies[10, 26, 27], the marginal zone of HAE lesions is an intense inflammatory reaction zone containing immune response cells (epithelioid cells, macrophages, lymphocytes, eosinophils, and other effectors cells, such as fibroblasts and myofibroblasts) and new blood vessels, which constitute the periparasitic granuloma at the border of the invaded liver parenchyma in AE. This region was approximately 1~2 cm wide. In addition, this reaction zone is also shown by imaging with contrast enhancement or focal FDG uptake in the perilesional hepatic parenchyma[8, 13, 14, 28–30]. Thus, the marginal zone was defined in CT images as an area less than 10 mm wide between the solid component of AE and the normal liver parenchyma (Fig 1). To ensure that the same lesion was measured at the same level byDECT, we maintained the size, shape, and location of the ROIs between the different scan phases and kept the measurement location in line. As shown in Fig 2, the display from the ROI placement includes the mean, standard deviation, minimum and maximum attenuation in HU, corresponding contrast-enhancement in HU (so-called “Overlay”), and IC in milligrams per milliliter (mg/ml) recorded. The actual ROI area ranged from 30 to 60 mm^2^. Three ROIs were drawn for each lesion, and the mean value of the 3 ROI measurements was used for further analysis.

### Histomorphological Analysis

The tissue sample point of the surgically removed gross HAE specimen was matched with the corresponding target ROIs on DECT images by marking sutures on the cranial, lateral, ventral, dorsal, medial and outside sites of the pathological specimen during the operation. All of the obtained HAE specimens were fixed in buffered formalin for at least 12 hours and then cut into 5-mm transverse slices to be consistent with the CT transverse plane. Then, the selected corresponding tissue blocks were embedded in paraffin and cut at 4-μm intervals for histological and immunohistochemical examination. Each block contained both part of the lesion and the surrounding normal liver parenchyma. The slides from each block were stained with hematoxylin-eosin (H&E) and Masson for routine diagnosis.

### Immunohistochemical Analysis

MVD counts were performed by immunohistochemistry with monoclonal antibodies to CD34 (CD34, polyclonal; BA0532, 1:50, Boster Biological Technology Co., Ltd., [Wuhan, China]) by a senior pathologist (P.D., with 8 years of experience in this field) who was blind to the clinical data and imaging results. Sections were scanned using a Moticam Pro 285A (Sony sensor, U.S.) for measurements. To count the microvessels, each section with CD34-stained tissue was first processed at low magnification (× 100) to spot three hot-spots representing the local areas of highest microvessel density, and then switched to high magnification (× 200) for clear imaging and better counting. For each slide, three hot-spot areas were counted, and the mean count of the large and small microvessels in each of the three hot-spot areas was the final MVD. The distribution of the hot-spot areas in the circular visual field of the sections was also recorded. The criteria for positive staining and microvessel count scoring system were as established by Sinicrope et al.[31]. The intensity of CD34 immunostaining was scored as follows: 0, no staining; 1+, weak (faint yellow); 2+, moderate (brown yellow); and 3+, intense (brown). The percentage of the total number of vessel cells was assigned to one of 5 categories: 0, ≤ 5%; 1, 5–25%; 2, 25–50%; 3, 50–75%; and 4, ≥ 75%. The percentage of positivity of the tumor cells and the staining intensity were multiplied to produce a weighted score for each specimen as a final score: 0, (-); 1–2, (+); 3–4, (++); and > 4 (+++). When the independent scoring of a case differed, the case was rechecked, and the final score was determined by recounting positive cells using a multi-headed microscope with each of the reviewers simultaneously viewing the slide.

### Statistical Analysis

The statistical analysis was performed with IBM SPSS Statistics version 20 (SPSS Inc, Chicago, Illinois). ICs and MVDs were compared between the groups. The fitness of the numeric dataset to normal distribution was determined using the Kolmogorov-Smirnov test.

Interreader reliability regarding DECT parameters was calculated using intraclass correlation coefficient analysis. The data were normally distributed, so the differences in ICs and MVDs between the different components of lesions and normal liver parenchyma and the three scan phases were analyzed using a univariate one-way analysis of variance (ANOVA) under the general linear model. A p value of less than 0.05 was considered statistically significant. To investigate the correlation between quantitative ICs and histopathologic parameter MVDs, linear correlations with Pearson’s correlation coefficient (SCC) were performed.

## Results

### DECT Findings

In 25 patients, 27 lesions were identified (solid type [n = 10], pseudocystic type [n = 3] and mixed type [n = 14]). The mean diameter of the HAE lesions was 100.7 ± 47.3 mm (range: 22–183 mm). Of these lesions, 16 were located solely in the right lobe of the liver, 6 were in the left lobe, and 5 involved both lobes. Intrahepatic dilation of bile ducts was found in 11 (44.0%) patients and vessel invasion in 16 (64.0%) patients. HAE lesions presented a mixed lower-density mass with a poorly demarcated border in the CT image, accompanied by unformed hyperattenuating calcifications and hypoattenuating regions related to necrosis. Among the 25 cases, three showed metastatis to other organs, including the lungs (2/25) and adrenal glands (1/25).

### Lesion Enhancement Pattern

**Fig 3:**
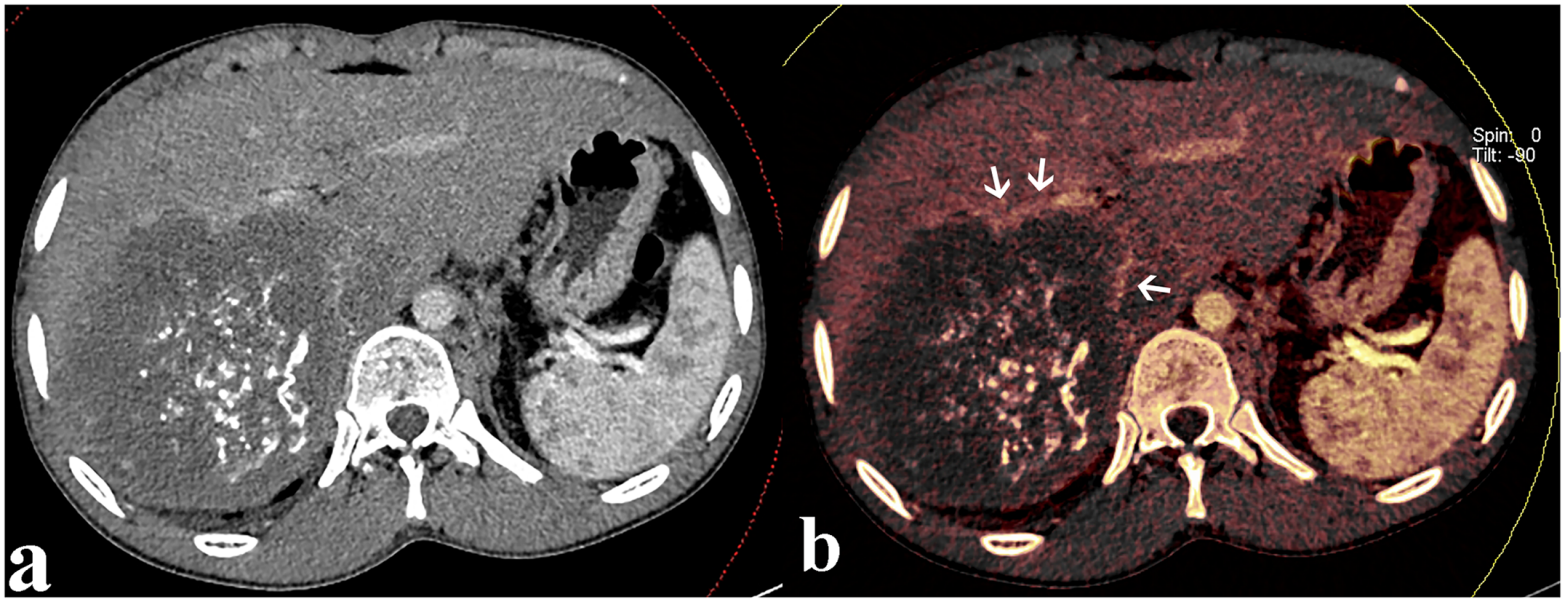
Example of image set of a patient with an AE on the right lober of liver. Two modes of images obtained by DECT: (a) conventional contrast enhanced CT images shows hypoattenuating lesion with center scattered hyperattenuating calcifications in the portal venous phase and faint enhancement of fibroinflammatory components surrounding the parasitic; (b) while this faint enhancement is more directly and strikingly on DECT contrast enhanced mixed iodine overlay images (arrowheads).

In 8/27 lesions, no enhancement was observed. In 19/27 lesions, a continuous or discrete circular rim-like enhancement and interval enhancement were visualized, especially during PVP and DP (52 ~ 90 s after the injection of contrast medium). Among these lesions, the mixed type accounted for 51.8% (14/27), the pseudocystic type accounted for 11.1% (3/27) and the solid type accounted for 7.4% (2/27). This finding was more precise in the iodine overlay image (Fig 3). The thickness of the enhanced belt ranged from 4 mm to 12 mm.

### Histopathologic Findings

**Fig 4:**
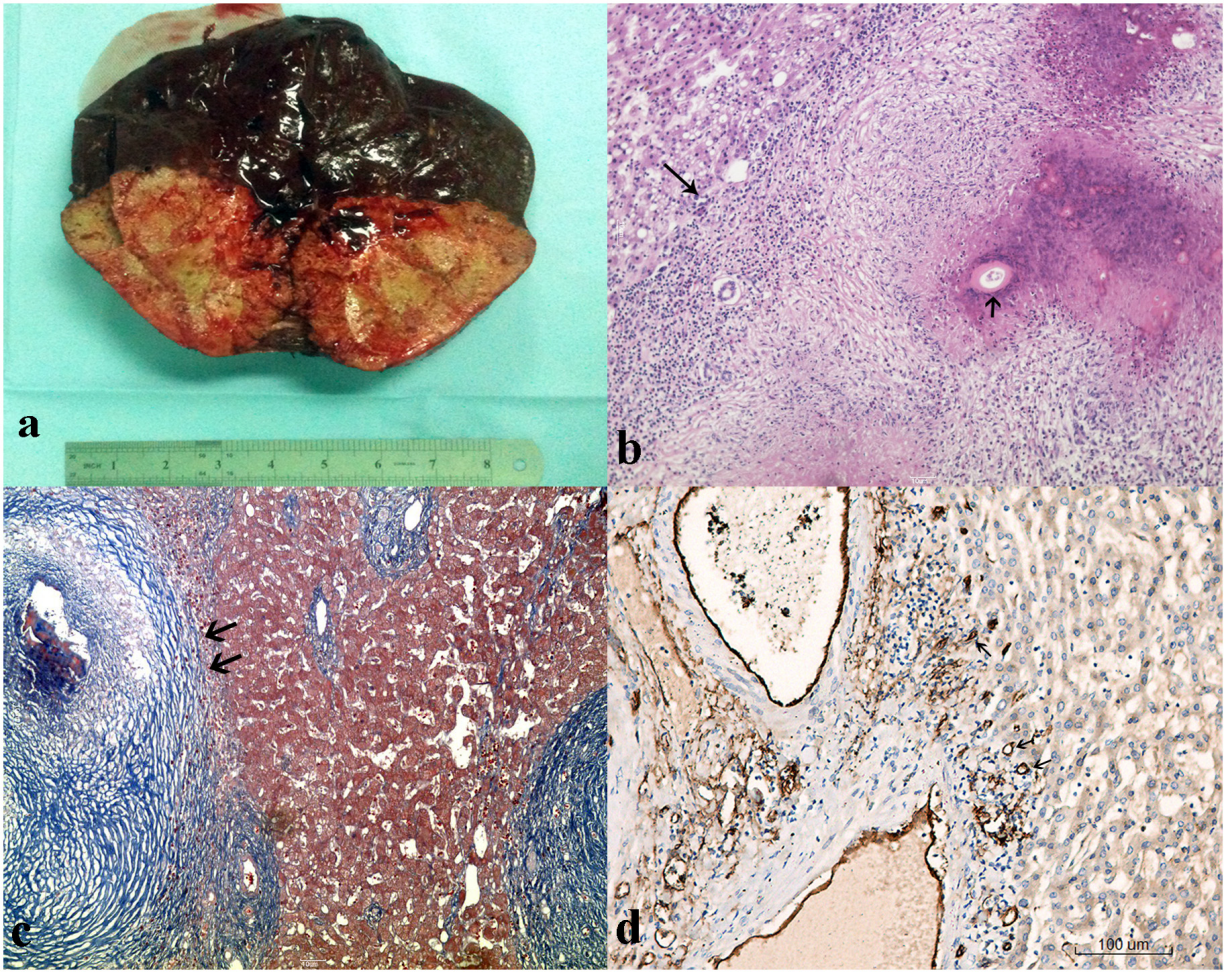
Macroscopical and the corresponding histopathologic views of HAE in a partial hepatectomy. (a) Photograph of a gross specimen enucleated from the liver shows a yellow infiltrative hard mass with no clear limits with the adjacent liver parenchyma. The periphery of this huge lesion composed of fibro-inflammatory tissue and small parasitic vesicles. (b) Photomicrograph (H-E staining, × 100) shows Brood capsule (arrowhead), inflammatory cell infiltration combine with stripe fibrous tissue and fatty degeneration (long arrow). (c) Photomicrograph (Masson staining, × 200) shows hyperchromatic blue stripe collagen fiber structure in the margin zone of HAE lesion (arrowheads). (d) CD34 antibodies (original magnification, × 200) shows large banding distribution brown microvascular structure in the margin zone of lesion (arrowheads). MVD = 28/HPF (0.72 mm^2^).

Upon microscopic analysis, H-E and Masson staining revealed the distribution of inflammatory cells (macrophages, lymphocytes and fibroblasts) around alveolar echinococcosis vesicles as a result of the immune response of the host, as characterized by homing to the adjacent liver (Fig 4b and 4c). Immunohistochemical staining of the microvessels at the marginal zone of HAE using CD34 showed a clear structure, and the integrated microvascular structure was rare. There was a large banding distribution of brown microvascular structures in the marginal zones of the lesions (Fig 4d). 92.5% (25/27) of HAE lesions showed positive expression of CD34 in the marginal zones, with 18.5% (5/27) strongly positive, 62.7% (17/27) moderately positive, 11.1% (3/27) weakly positive, and 7.4% (2/27) negative. In normal liver tissues, 25.9% (7/27) of samples showed positive expression of CD34. There was no strongly positive staining, with 3.7% (1/27) moderately positive, 22.2% (6/27) weakly positive, and 74% (20/27) negative staining.

### Comparison of IC and MVD

Interreader reliability regarding DECT parameters was high and significant, with intraclass correlation coefficients of 0.90 to 0.96 (P < 0.05 each) for measurements in the margin zone cross-section ROI, of 0.77 to 0.85 (P < 0.05 each) in the solid site crosssection ROI, of 0.79 to 0.89 (P < 0.05 each) in the cyst area ROI, and of 0.81 to 0.89 (P < 0.05 each) in the liver parenchyma ROI. Because of the high interreader reliability, the measurements of only 1 reader (ie, the first reader) were used for further analyses.

The results of the quantitative IC and MVD counts are summarized in Table 1. The differences in the IC and MVD count between marginal zones, solid and cyst components of HAE lesions and normal liver parenchyma were significant for all of the contrast-enhanced scan phases. The mean IC of the marginal zone was higher than that of the solid and cyst components in the AP, PVP and DP. Meanwhile, the mean ICs of the marginal zone were higher in PVP and DP than in AP. The IC parameters for different types of HAE lesions are reported in Table 2. No significant difference was found in the mean IC of the marginal zone among HAE lesions with different CT patterns. There was a significant increase in the vascular parameters of MVD in the marginal zone of HAE lesions compared to inner HAE lesions and normal liver tissue. The MVD count was low in inner HAE lesions.

**Table 1:** IC measurements and MVD counts in HAE lesions.

Lesion component	IC (mg/ml)			MVD (vessels/HPF)
	AP	PVP	DP	
**Marginal zone**	2.15 ± 0.71	3.08 ± 0.85	2.93 ± 0.77	25.66 ± 0.98
**Solid**	0.17 ± 0.09	0.10 ± 0.08	0.07 ± 0.07	0.66 ± 0.91
**Cyst**	0.02 ± 0.04	0.03 ± 0.06	0.01 ± 0.01	0.00
**Normal liver**	1.35 ± 0.29	2.04 ± 041	1.86 ± 0.35	5.60 ± 1.30
**P**	< 0.01	< 0.01	< 0.01	< 0.01

IC = iodine concentration, AP = artery phase, PVP = portal venous phase, DP = delayed phase, MVD = microvessel density

**Table 2:** Quantitative assessment of IC parameters for types of HAE lesions*.

IC in Marginal zone	Solid type (n = 10)	Pseudocystic type (n = 3)	Mixed type (n = 14)	P-value
**AP**	1.72 ± 0.56	2.50 ± 0.42	2.41 ± 0.75	0.06
**PVP**	2.62 ± 0.72	3.75 ± 0.91	3.40 ± 0.90	0.08
**DP**	2.50 ± 0.60	2.00 ± 0.28	3.18 ± 0.84	0.08

IC = iodine concentration, AP = arterial phase, PVP = portal venous phase, DP = delayed phase.

*According to the classification system of Merkle E et.al. [25]

### Correlation between DECT and Histopathologic Parameters

The correlations between IC and MVD for different components and types of HAE lesions are summarized in Table 3. Pearson’s correlation analysis revealed an association between the IC and MVD count in AP (r = 0.425; p = 0.027), PVP (r = 0.731, p<0.001) and VP (r = 0.712, p < 0.001) for the marginal zone of HAE lesions and in AP (r = 0.514; p = 0.006), PVP (r = 0.556, p = 0.003) and VP (r = 0.488, p = 0.01) for normal liver parenchyma. There was no correlation between the IC and MVD count for the solid component or cyst component of HAE lesions in any of the contrast scan phases. Furthermore, there was a statistically significant positive correlation between the IC and MVD for the corresponding marginal zone of mixed-type HAE lesions. The correlation coefficients (r) were 0.59 (P = 0.026) for PVP and 0.61 (P = 0.018) for DP. However, MVD had no positive correlation with IC in solid–type lesions. No statistical analysis was performed for the correlation between IC and MVD in the pseudocystic type because there was an insufficient number of cases (n = 3).

**Table 3:** Correlations between IC and MVD from different components and types of HAE lesions.

MVD	IC (mg/ml)		
	AP	PVP	DP
**Margin zone**	0.42*	0.73**	0.71**
**Solid**	-0.06^	-0.05^	-0.37^
**Liver Parenchyma**	0.51**	0.55**	0.48*
**Mixed type**	0.36^	0.59*	0.61*
**Solid type**	0.23^	0.31^	0.32^

IC = iodine concentration, AP = arterial phase, PVP = portal venous phase, DP = delayed phase, MVD = microvessel density, Correlation is significant at ^P>0.05, *P<0.05 and **P<0.01.

## Discussion

We report here the utilization of quantitative IC of DECT for the assessment of HAE vascularization. Our results reveal that DECT imaging based on the differentiation of iodine quantification is technically feasible and improves HAE vascularity detectability and lesion conspicuity compared to those of the conventional CT system. Furthermore, in this study histopathological microvascular parameters were compared with DECT pharmacokinetic parameters, and we found a positive correlation between the IC and MVD count in the marginal zone of HAE lesions. This preliminary result indicates that iodine concentration measurement using DECT imaging can quantitatively identify the microperfusion status of the periparasitic granulomatous reaction and can indirectly, albeit based on a different pathological substratum, reflect the activity of AE lesions.

For years, the vascularization or contrast enhancement pattern of AE has been controversial. From early observation of lacking of both intralesional and rim enhancement to peripheral vascular structures detection of echinococcal lesion in many imaging studies[25, 27, 29, 32–34]. These data contradict findings indicating methodical advances in imaging techniques and improvements in diagnostic accuracy. In our study, more than half of the lesions (70%, 19 of 27) were visualized by peripheral or intralesional contrast enhancement, especially in the portovenous phase and delayed phase of scanning (from 52–90 s). These findings to some extent are consistent with those of Ehrhardt et al.[29] and Coskun et al.[28], which showed peripheral enhancement of lesions in the late contrast-enhancement phase but found that the detection rate was higher on the iodine overlay image. However, these studies lacked quantitative parameters and could not allow comparisons with histopathologic parameters for interpretation. The problem of whether CT can provide reliable information regarding the viability of the parasitic lesion has remained unsolved until now.

Unlike traditional CT imaging, which can reveal only the morphology and density of pathological lesions, DECT is a new approach for functional imaging that improves material differentiation and achieves quantitative analysis[17]. In contrast-enhanced DECT, the contrast medium is carried into the lesion by new, viable blood vessels, and quantitative IC is derived from a pharmacokinetic model analysis of DECT based on the mathematical model that is used. Under a relatively fixed concentration and flow rate of the iodinated contrast medium, the IC should be proportional to the blood perfusion in segmented lesions[16–18] and thus reflect the vascularity of the focal lesion. This relationship highlights the idea that IC could be a promising tool for evaluating blood supply status in HAE lesions. In this study, we calculated quantitative DECT variables in different tissues (marginal zone, solid and cystic) of HAE lesions and found that IC was significantly higher in the marginal zone of an HAE lesion than in the solid or cystic components; the IC measurements were significantly different between the marginal zone, cystic component and solid component of HAE lesions in all three of the contrast-enhanced scan phases, as shown in Table 1. This result confirms the existence and distribution of vascular micro-perfusion of HAE lesions and demonstrates that IC is likely to serve as a valuable functional imaging parameter for evaluating vascularity in HAE lesions. Moreover, we found that IC measurements from the marginal zone in PVP were higher than in VP or AP, and there was a significant difference in IC between AP and PVP but no significant difference between PVP and VP. Similar findings were reported by Ehrhardt, A. R. et al.[29] in angiographic studies of 17 patients with AE; the authors observed that contrast enhancement occurred at the margins of the majority of the lesions (77.8%, seven of nine) during the parenchymal-portovenous phase (>40 s after injection of the contrast medium). These findings might suggest that the gross enhancement pattern of HAE lesions with delayed enhancement is related to the microcirculation that is dominant within an HAE lesion. In addition, we found that the peripheral contrast enhancement of HAE lesions can be screened easily in the PVP rather than VP, which might be explained by the low contrast between liver parenchyma and lesions in the VP.

Angiogenesis is an important part of the cell-mediated immune reaction in periparasitic granulomas. Neovascularization indicates that a complex response may be be involved in the metastatic process that is observed in AE[10]. MVD, although not regarded as the best biological marker for the viability of HAE, is more appropriate for the image index based on vascular characteristics and hemodynamic changes and as an indirect measure of AE progression. In fact, the correlation between CT perfusion parameters and the extent of tumor angiogenesis has been investigated in various tumors[35–37] and has shown significant correlations with MVD. However, few previous studies have studied the correlation between histopathological microvascular parameters and DECT in AE. To the best of our knowledge, the current study is the first to use DECT in HAE and to investigate the usefulness of IC for evaluating the angiographic vascularity of HAE lesions.

Our study revealed a positive correlation between the IC and the angiogenic MVD count in the marginal zone of HAE lesions, and there was no correlation between the IC and MVD count in the solid component or the cyst component. However, the histomorphological distribution characteristics of microvessels might be implicated in HAE lesions, meaning that blood distribution exists mainly around the HAE lesion. There were few IC (0.00–0.3 mg/mL) measurements and MVD counts (0.00–3 vessels/HPF) in the solid and cyst components of HAE lesion, and no intact vascular structures were found in CD34-expressing regions In addition, the contrast medium uptake focused primarily on the perilesional area of HAE lesions, which is consistent with the location of contrast enhancement in imaging studies. However, because the MVD counts represented the hemodynamic characteristics of lesion microvasculature, this result indicates that IC could truly reflect the distribution of microvessels of HAE on the pathological substratum, as the areas with higher IC matched the areas with high MVD. Furthermore, because increased IC is associated with significant enhancement of focal lesions, the degree of lesion enhancement on DECT is affected by MVD. This result means that the enhancement degree reflects the distribution characteristics of lesion microvessels, as the area with the greatest enhancement was often associated with the highest MVD. Therefore, from a clinical perspective, by analyzing the enhancement morphology of HAE lesions on CT, it is possible to provide in vivo overall functional information about angiogenesis, in addition to accurate morphological information.

In the second part of the study, we tried to correlate ICs with MVDs in HAE lesions with different CT patterns, as defined by Merkle, E et al.[25]: solid type (CT images showing a homogenous hypodense lesion without any visible liquid component); pseudocystic type [CT images showing a predominantly large cystic lesion with the density of water (+5 to +10 Hounsfield Units) with a ragged necrotic wall]; and mixed type (geographic map) (CT images showing heterogeneous lesions with both solid and liquid components in varying proportions appearing like a geographical map). It is known that this CT-based morphological classification scheme reflects disease pathophysiology in AE to some extent. Our findings showed a positive correlation between the IC and MVD of the corresponding marginal zone of mixed-type HAE lesions but not in solid-type lesions. No statistical analysis was performed for the correlation between IC and MVD in pseudocystic type lesions because there was an insufficient number of cases (n = 3). In addition, we subsequently compared the ICs of the marginal zones among the three types of HAE lesion (based on CT imaging) and found no significant differences. Statistically, however, the IC of the marginal zones of pseudocystic lesions was higher than that of the mixed and solid type in the PVP (3.75 ± 0.91 mg/ml vs. 3.40 ± 0.90 mg/ml, 2.62 ± 0.72 mg/ml). These findings were expected because the pseudocystic type or mixed type, which contains more liquefaction necrosis, has been associated with the developing stage[38] and is likely to become co-infected with other bacteria and fungi, possibly leading to complications such as liver abscesses. For most pseudocystic hepatic lesions, CT shows a large necrotic central area that is surrounded by an irregular ring-like region of fibroinflammatory tissue that often forms a faint enhancement, most likely due to the accumulation of contrast agent in the underlying fibrotic component[6]. These image findings resemble hepatic abscess cavities.

There are some potential limitations that must be considered. First, the number of patients in our study is relatively small because HAE is a rare disease. Moreover, small numbers of pseudocystic type (n = 3) lesions were evaluated compared to the other lesion types (n ≥ 10). Therefore, additional studies with a larger number of patients need to be performed for validation. Second, there was no precise correlation between IC in DECT and pathologic specimens after surgery. The IC assessment criterion has not yet been established. Further quantitative studies should correlate IC with other independent parameters, such as the image index of functional quantitative glucose metabolism on FDG-PET, to more appropriately define IC thresholds for the monitoring of HAE-targeted therapy.

## Conclusion

This study showed that iodine mapping using DECT imaging can be used to visualize and quantify the vascularization of typical patterns of HAE lesions, providing a distinct overview of the spatial distribution of microvascular characteristics. The IC measurement is easy, robust, and less observer-dependent than other methods, and is therefore likely to serve as a valuable functional imaging parameter for measuring perfusion and lesion vascularity. Thus, the measurement of IC with DECT could be a promising predictor of HAE progression.

## Supporting Information

S1 Table Relevant data underlying the findings described in manuscript. (XLSX)
